# Computed tomographic findings in 25 cats with ear canal neoplasia

**DOI:** 10.1111/vru.13467

**Published:** 2024-12-16

**Authors:** Megan Wisnoski, Christine Gremillion, Gwendolyn Levine, Cambridge Coy, Kaylynn Veitch, Kenneth Waller, John F. Griffin

**Affiliations:** ^1^ Department of Large Animal Clinical Sciences College of Veterinary Medicine and Biomedical Sciences Texas A&M University College Station Texas USA; ^2^ Department of Surgical Sciences University of Wisconsin Madison Wisconsin USA

**Keywords:** aural, benign, malignant, neoplasm

## Abstract

Computed tomography is commonly used to evaluate feline otic disease; however, published studies characterizing the CT appearance of ear canal neoplasia are limited. The purpose of this multicenter, retrospective, secondary analysis, cross‐sectional study was to describe the CT features of histopathologically confirmed feline ear canal neoplasia. The CT studies of 25 cats with ear canal neoplasia were prospectively scored by consensus of two veterinary radiologists. Recorded parameters were the presence of focal or multifocal tissue enlargement (mass/masses), lesion shape, location of the center of mass, attenuation characteristics, features of contrast enhancement, involvement of otic structures, calvarial and brain changes, changes of nearby structures, and lymphadenopathy. There was a significant overlap of CT findings between cats with malignant ceruminous gland neoplasia, ceruminous gland adenoma, and squamous cell carcinoma (SCC). Ceruminous gland adenoma was typically homogeneous in attenuation with homogeneous contrast enhancement and no intralesional fluid accumulations (IFAs) or involvement of adjacent structures. In contrast, SCC consistently had heterogeneous attenuation, heterogeneous contrast enhancement, IFAs, and involvement/invasion of adjacent structures. Malignant ceruminous gland neoplasia had variable attenuation and pattern of contrast enhancement with occasional IFAs and occasional involvement/invasion of adjacent structures. Knowledge of these imaging features will inform the creation of prioritized differential diagnosis lists. However, a biopsy is required to confirm the diagnosis.

## 1 INTRODUCTION

Feline ear canal neoplasia is relatively uncommon in comparison to the incidence of general otic disease. Ear canal tumors account for 1–2% of all tumors in cats and exhibit a high probability of malignancy, exceeding 80%.^1^, ^2^, ^3^ In cats, ceruminous gland adenocarcinoma and squamous cell carcinoma (SCC) are the most common malignant neoplasms in the ear canal and middle ear, respectively.^1^, ^4^, ^5^, ^6^ Various types of malignant ear canal neoplasms have been documented in veterinary literature, including ceruminous gland carcinoma, sebaceous gland adenocarcinoma, carcinoma of undetermined origin, and lymphoma.^2^, ^7^, ^8^ Ceruminous gland adenoma (CGA) is the most frequently occurring benign aural neoplasm in cats.^4^, ^9^ However, several other tumor types, including sebaceous gland adenoma, basal cell tumors, histiocytomas, papillomas, and fibromas, have also been reported.^10^ Feline inflammatory polyps are the most common non‐neoplastic masses in the external ear canal.^6^, ^11^, ^12^

Computed tomography is commonly utilized in the evaluation of feline otic disease. CT is used to narrow the differential diagnosis list, identify sites for tissue sampling, determine disease extent (for surgical planning), identify metastatic disease, and evaluate response to therapy.

Currently, there are limited reports describing the CT imaging characteristics of ear canal tumors in cats.^1^, ^4^, ^5^ Knowledge of the span of CT findings will improve veterinary care by raising clinical suspicion and guiding additional diagnostics and therapeutic intervention. Therefore, the objective of this study was to describe the CT features of histologically confirmed ear canal neoplasia in a population of cats.

## 2 MATERIAL AND METHODS

This was a multicenter, retrospective, secondary analysis, cross‐sectional study design. Medical records at Texas A&M University (TAMU) and the University of Wisconsin (UW) were searched for client‐owned cats with histopathologically confirmed neoplasia of the vertical ear canal, horizontal ear canal, middle ear, and inner ear and contrast‐enhanced CT scans of the lesion. Cats with pinna lesions were not included. Cases of known inflammatory processes and neoplastic cases with only cytologic confirmation were excluded. As this was a retrospective study, institutional animal care and use committee approval were not needed. Each hospital director approved the use of hospital data for this study.

Clinical data were extracted from the medical records by licensed veterinarians (C.C. and K.V.) and third‐year veterinary student (M.W.). The following data were recorded: contributing institution, age (years), sex (male, castrated male, female, spayed female), breed, body weight (kilograms), date of CT, and date of histopathological acquisition. As this was a retrospective study, the CT acquisition protocol was not standardized. Based on histopathologic diagnosis, cases were assigned to four groups: malignant ceruminous gland neoplasia (MCGN), CGA, SCC, and other.

CT scans were performed on various CT systems per each institution's protocols. Specific technical parameters were not recorded. CT studies were reconstructed using a variety of algorithms (e.g., bone, soft tissue) and displayed in soft tissue and bone window width and level. All CT studies were reviewed by two ACVR‐certified radiologists (C.G. and G.L.), and findings were recorded by the primary author (M.W.) based on consensus using a predesigned data collection form (Table S1). Images were evaluated using DICOM viewing software (eUnity 7.0 and IntelliSpace). Evaluators reviewed the entire study for all cases, and, at a minimum, all cases were evaluated in a soft tissue and bone window width and level in the transverse plane. When available, additional reconstructions were reviewed (e.g., sagittal and dorsal plane reformats). Evaluators had access to the entire study for all cases, and all cases were evaluated using a soft tissue algorithm. Evaluators were aware that the final diagnosis was ear canal neoplasia but unaware of the specific type of neoplasia. Evaluators were permitted to adjust the window width and window level. Interpretations were recorded, instances of initial discrepancy between reviewers were promptly discussed, and a final designation was reached by consensus agreement. Manuscript reporting was based on the STROBE guidelines.^13^

A mass was defined as the subjective perception of tissue enlargement. The number of masses and the side where tissue enlargement was the most severe were recorded. In cases with multifocal disease, the analysis was then conducted based on the largest mass. In short, masses were evaluated for shape (round/oval broad‐based vs. round/oval pedunculated vs. plaque‐like vs. amorphous), location (vertical ear canal, horizontal ear canal, middle ear, inner ear), attenuation characteristics, features of contrast enhancement (diffusely heterogeneous vs. heterogeneous with peripheral enhancement vs. homogeneous vs. none), involvement of otic structures (displacement, compression, invasion, destruction), calvarial and brain changes (periosteal proliferation, osteolysis, foraminal enlargement, meningeal thickening and enhancement, and intracranial invasion), and changes of nearby structures (lymph nodes, parotid salivary gland, skeletal muscle, and pharynx). Osteolysis was defined as the destruction of normal cortical or trabecular bone architecture. Amorphous, sunburst, spiculated, and palisading periosteal proliferation were considered aggressive.^5^ Smooth and lamellar periosteal proliferation was considered nonaggressive.

## 3 RESULTS

### 3.1 Cases

The study population consisted of 25 cats from TAMU (*n* = 19) and UW (*n* = 6). Computed tomographic imaging was performed between 2007 and 2022.

### 3.2 Demographics

The MCGN group included 14 cats. The median age was 12 years (range, 6–17 years). The median weight was 5.3 kg (range, 2.9–7.9 kg). There were 11 castrated male cats and 3 spayed female cats. There were 11 domestic shorthairs, 1 domestic medium hair, 1 domestic longhair, and 1 Persian. The median time from CT to histopathological sample acquisition was 1.5 days (range, 0–21). The CGA group had six cats. The median age was 10.5 years (range, 7–15 years). The median weight was 4.4 kg (range, 2.7–5.8 kg). There were four castrated male cats and two spayed female cats. There were four domestic shorthairs, one domestic longhair, and one Siamese. The median time from CT to histopathological sample acquisition was 1.5 days (range, 0–8). The SCC group included three cats. The median age was 8 years (range, 7–12 years). The median weight was 3.6 kg (range, 2.1–3.9 kg). All three were spayed female cats. There were two domestic shorthairs and one Siamese. The median time from CT to histopathological sample acquisition was 0 days (range, 0–21). The group with other types of neoplasia included two cats: papillary adenoma (16‐year‐old castrated male, domestic shorthair, 5.1 kg) and adenocarcinoma (5‐year‐old castrated male, domestic medium hair, 6.4 kg). The time from CT to histopathological sample acquisition was 0 days for both cases.

### 3.3 CT findings

The CT findings are summarized in Tables 1 and 2. All cats with a mass present (*n* = 24) had soft tissue attenuating, contrast‐enhancing masses. The contrast enhancement was either diffusely heterogeneous (*n* = 15) or homogeneous (*n* = 9). All masses were located in the external ear canal.

**TABLE 1:** CT findings in cats with ear canal neoplasia.

	Malignant ceruminous gland neoplasia (*n *= 14)	Ceruminous gland adenoma (*n *= 6)	Squamous cell carcinoma (*n *= 3)
**Number**			
Focal mass	11	5	3
Multifocal unilateral masses	1		
Multifocal bilateral masses	1	1	
No mass	1		
**Shape**			
Round/ovoid shape	6	2	1
Pedunculated	1		
Plaque‐like	2	2	
Amorphous/Other	5	2	2
**Location**			
Vertical ear canal	7	2	
Horizontal ear canal	3	3	2
Middle ear			
Inner ear			
UTD	3	1	1
NA	1		
**Attenuation characteristics**			
Homogeneous	7	5	
Heterogeneous	6	1	3
Intralesional gas			1
Intralesional fat			
Intralesional mineral	3	2	2
Intralesional fluid	3		3
**Contrast enhancement**			
Diffusely heterogeneous	10	2	3
Peripherally enhancing			
Homogeneous	3	4	
Nonenhancing			
**Other**			
Osteolysis			3
Skull foraminal enlargement	1		1
Aggressive PP	1		2
Nonaggressive PP			
Meningeal changes			1

Abbreviations: NA, no mass identified; PP, periosteal proliferation; UTD, unable to determine/categorize.

**TABLE 2:** Adjacent/regional structure involvement in cats with ear canal neoplasia.

	Malignant ceruminous gland neoplasia (*n *= 14)	Ceruminous gland adenoma (*n *= 6)	Squamous cell carcinoma (*n *= 3)
**LN enlargement**			
Parotid	9	2	
Lateral retropharyngeal	13	3	2
Medial retropharyngeal	13	2	3
Mandibular	5	2	
Superficial cervical^a^	6/9	1/2	1/1
Deep cervical^a^	3/10	0/2	0/1
**Parotid salivary gland**			
Compression/displacement	4		2
Invasion/destruction			1
Combination of the above	1		
**Surrounding musculature**			
Compression/displacement	3		
Invasion/destruction			1
Atrophy			
Combination of the above			1
**Pharynx**			
Compression/displacement			2
Invasion/destruction			
Combination of the above			

^a^ The superficial cervical and deep cervical lymph nodes were not included in the field‐of‐view for all studies.

Abbreviations: LN, lymph node; SCC, squamous cell carcinoma.

**FIGURE 1:**
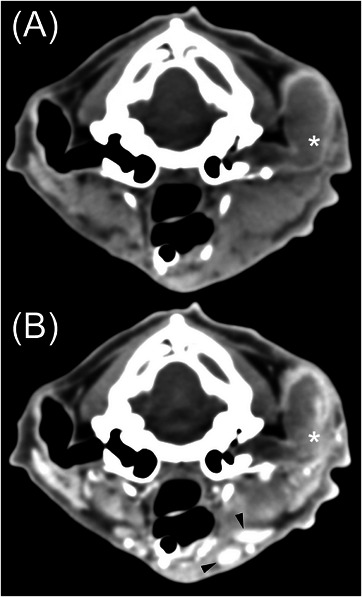
Twelve‐year‐old castrated male domestic shorthair with a malignant ceruminous neoplasm. A, Precontrast soft tissue transverse CT image. The left external ear canal is filled with soft tissue and fluid‐attenuating material. B, Postcontrast soft tissue transverse CT image. This mass (asterisks) has homogeneous attenuation, moderate to marked, heterogeneous contrast enhancement, an irregular luminal margin, and shares a broad margin with the vertical ear canal wall.

The MCGN group had 14 cats (see Figure 1). Most cats had a focal lesion (*n* = 11), while two cats had multifocal masses, and one cat had no identified lesion. All masses were soft tissue attenuating. Attenuation was rated as homogeneous (*n* = 7) or heterogeneous (*n* = 6). Three cases had intralesional mineral, and 3 had intralesional fluid. Contrast enhancement was rated as diffusely heterogeneous (*n* = 10) or homogeneous (*n* = 3). All cats had some regional lymphadenopathy, with lateral and medial retropharyngeal enlargement being the most common (*n* = 13), followed by parotid lymph node enlargement (*n* = 9). One cat had skull foraminal enlargement of the left oval foramen, and another had aggressive periosteal proliferation (on the lateral aspect of the tympanic bulla).

**FIGURE 2:**
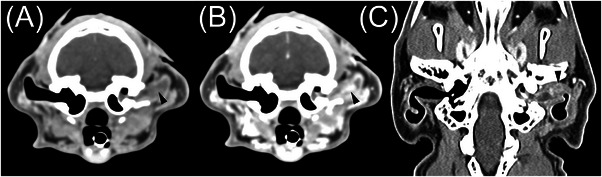
Fifteen‐year‐old castrated male domestic shorthair with a ceruminous gland adenoma. A, Precontrast soft tissue transverse CT image. B, Postcontrast soft tissue transverse CT image. C, Postcontrast soft tissue dorsal CT image. This mass (black arrowheads) has homogeneous attenuation, homogeneous contrast enhancement, well‐defined margins, and an ovoid shape. It shares a broad margin with the wall of the horizontal ear canal. The left horizontal ear canal contains a mild volume of fluid‐attenuating, noncontrast‐enhancing material.

The CGA group had 6 cats (see Figure 2). Most cats had a focal lesion (*n* = 5), while one cat had multifocal masses. All masses were soft tissue attenuating. Attenuation characteristics were rated as homogeneous (*n* = 5) or heterogeneous (*n* = 1). Two cats had intralesional mineral. Contrast enhancement was rated as diffusely heterogeneous (*n* = 2) or homogeneous (*n* = 4). All masses were located in the external ear canal.

**FIGURE 3:**
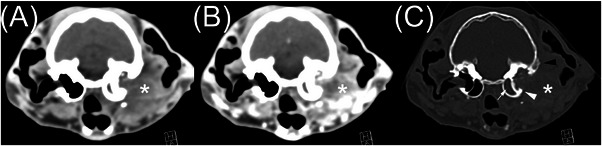
Eight‐year‐old spayed female Siamese with a squamous cell carcinoma. A, Precontrast soft tissue transverse CT image. B, Postcontrast soft tissue transverse CT image. C, Postcontrast bone window transverse CT image. This mass (asterisk) has heterogeneous attenuation, heterogeneous contrast enhancement, and indistinct margins. There is lysis of the temporal bone (white arrowhead), aggressive periosteal proliferation of the temporal bone (black arrowhead), and tympanic cavity fluid and/or soft tissue (small white arrow).

**FIGURE 4:**
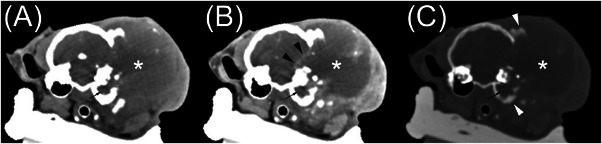
Seven‐year‐old spayed female domestic shorthair with a squamous cell carcinoma. A, Precontrast soft tissue transverse CT image. B, Postcontrast soft tissue transverse CT image. C, Postcontrast bone window transverse CT image. There is a large mass of the left external ear canal (asterisk). The mass has a large intralesional fluid accumulation, multifocal mineral attenuation, and heterogeneous contrast enhancement. There is a large osteolytic calvarial defect and invasion of the rostral fossa with meningeal thickening, contrast enhancement (black arrowheads), and a mass effect with a shift of the falx cerebri to the right and compression of the left lateral ventricle. There is an aggressive periosteal reaction on the temporal and parietal bones (white arrowheads) and tympanic cavity fluid and/or soft tissue (small black arrow).

The SCC group had three cats (see Figures 3 and 4). Involvement of the middle ear (*n* = 3) and inner ear involvement (*n* = 1) was observed. All masses were soft tissue attenuating. Attenuation was rated as heterogeneous (*n* = 3). One had intralesional gas, two had intralesional mineral, and all three had intralesional fluid. Contrast enhancement was rated as diffusely heterogeneous (*n* = 3). Other important findings included osteolysis (*n* = 3), aggressive periosteal proliferation (*n* = 2), skull foraminal enlargement (*n* = 1) of the right orbital fissure, round foramen, oval foramen, internal acoustic meatus, facial canal, and stylomastoid foramen, and meningeal changes (*n* = 1). The osteolysis involved the temporal bone (near the external acoustic meatus) in all three cases and the adjacent occipital and parietal bone in one case. The aggressive periosteal proliferation involved the temporal and adjacent occipital bones in two cases, as well as the parietal bone in one of these cases. The meningeal contrast enhancement was located adjacent to a large osteolytic defect in the temporal and parietal bone.

The other neoplasia group had one cat with papillary adenoma and one with adenocarcinoma. Both were solitary masses that were homogeneous in attenuation with a homogeneous pattern of contrast enhancement.

## 4 DISCUSSION

The purpose of this study was to characterize the CT features of histopathologically confirmed neoplasia in the ear canal of cats and to identify any distinctive CT features for each type of neoplasia. The study's results showed a significant overlap of CT findings between MCGN, CGA, and SCC. All three main groups had typically solitary lesions with varying shapes. No pattern was observed regarding horizontal or vertical ear canal location in benign versus malignant neoplasia. Intralesional mineral attenuation and lymphadenopathy were also seen in all three main groups. These results of overlap between benign and malignant neoplasia are consistent with previous reports of GI, oral, and gastric tumor types.^14^, ^15^, ^16^ That said, our findings suggest that a few characteristics may help distinguish benign from malignant feline ear canal neoplasia, with the understanding that they should not be relied upon individually to determine prognosis.

Most cases of CGA, as demonstrated by Figure 2A, had homogeneous attenuation, compared with only about half of the MCGN cases and none of the SCC cases. In addition, none of the CGA cases had intralesional fluid accumulations, compared with a few of the MCGN cases and all of the SCC cases. We suggest that heterogeneous attenuation, as demonstrated in Figures 3A and 4A, increases the likelihood of malignancy. Heterogeneous attenuation in precontrast images is a CT feature reported in other malignant soft tissue tumors.^17^, ^18^, ^19^

The majority of CGA cases had homogeneous contrast enhancement compared with only a few MCGN cases and none of the SCC cases. We suggest that homogeneous contrast enhancement increases the likelihood of CGA. Our results align with previous studies of other types of tumors, showing that lesional post‐contrast enhancement patterns were found to be strong indicators of malignancy.^15^, ^17^, ^18^, ^19^, ^20^ Our findings highlight the importance of utilizing contrast medium in CT scans.

In this study, there were no cases of CGA with osteolysis, skull foraminal enlargement, periosteal proliferation, meningeal changes, or involvement of the parotid salivary gland, surrounding musculature, or pharynx. In contrast, skull foraminal enlargement (*n* = 1), aggressive periosteal proliferation (*n* = 1), parotid salivary gland compression/displacement (*n* = 4), parotid salivary gland invasion (*n* = 1), and compression/displacement of surrounding musculature were occasional findings in MCGNs. SCC frequently had osteolysis (*n* = 3), skull foraminal enlargement (*n* = 1), aggressive periosteal proliferation (*n* = 2), meningeal changes (*n* = 1), parotid salivary gland compression/displacement (*n* = 2), parotid salivary gland invasion (*n* = 1), compression/displacement of surrounding musculature (*n* = 2), invasion of surrounding musculature (*n* = 2), and compression/displacement of the pharynx (*n* = 2).

Computed tomographic imaging features of inflammatory polyps and otitis externa have been described.^21^, ^22^, ^23^ Both neoplastic lesions and inflammatory polyps tend to exhibit contrast enhancement, but inflammatory polyps often have only strong rim enhancement.^21^ ^,23^ Lysis of the tympanic bulla and soft tissue proliferation resulting in luminal stenosis and obstruction of the external ear canal can be seen with neoplasia and chronic otitis externa.^22^ While there is a significant overlap of the spectra of imaging features between neoplasia and chronic otitis externa, the magnitude of soft tissue enlargement in neoplasia is probably larger, and the distribution is probably more likely to be focal rather than diffuse. Furthermore, there are some distinguishing characteristics of otitis externa, such as mineralization of the ear canal and marked ceruminous gland hypertrophy and hyperplasia.^22^ It is important to note that many cats have concurrent otitis and ear canal neoplasia.

There are a few strengths of this study. First, we were able to increase the number and diversity of cases by enlisting a multicenter approach and including cases over 15 years. Second, the CT studies were acquired at different institutions and varied in acquisition parameters. This could make our results more applicable to the wider veterinary imaging community. Lastly, all cases had a histopathological diagnosis of ear canal neoplasia.

There were a few limitations to the study. Specific technical parameters used for CT studies were not recorded. Whether these technical parameters could have affected imaging characteristics, therefore, remains unknown. The requirement for histopathological diagnosis probably biased the population toward cats with more severe diseases. The SCC group was very small in this study, and findings may not be truly representative of the population. Finally, this study was not designed to investigate the diagnostic accuracy or repeatability of CT or factors related to outcome. We hope that the information provided herein will be helpful to others in the generation of hypotheses for future prospective studies.

## 5 CONCLUSION

In our multicenter sample of cats, aural neoplasia exhibited some characteristic CT features with a wide range of variation. There was a significant overlap of CT findings between cats with MCGN, CGA, and SCC. CGA was typically homogeneous in attenuation with homogeneous contrast enhancement and no IFAs or involvement of adjacent structures. In contrast, SCC consistently had heterogeneous attenuation, heterogeneous contrast enhancement, IFAs, and involvement/invasion of adjacent structures. MCGN had variable attenuation and pattern of contrast enhancement with occasional IFAs and occasional involvement/invasion of adjacent structures. Knowledge of these imaging features will inform the creation of prioritized differential diagnosis lists. However, a biopsy is required to confirm the presumptive diagnosis.

## LIST OF AUTHOR CONTRIBUTIONS

### Category 1

(a) Conception and design: Coy, Griffin, Waller
(b) Acquisition of data: Coy, Wisnoski, Gremillion, Levine, Veitch, Waller
(c) Analysis and interpretation of data: Coy, Wisnoski, Gremillion, Levine, Griffin, Veitch, Waller

### Category 2

(a) Drafting the article: Wisnoski, Griffin
(b) Revising article for intellectual content: Coy, Wisnoski, Gremillion, Levine, Griffin, Veitch, Waller

### Category 3

(a) Final approval of the completed article: Coy, Wisnoski, Gremillion, Levine, Griffin, Veitch, Waller

### Category 4

(a) Agreement to be accountable for all aspects of the work in ensuring that questions related to the accuracy or integrity of any part of the work are appropriately investigated and resolved: Coy, Wisnoski, Gremillion, Levine, Griffin, Veitch, Waller

## CONFLICT OF INTEREST STATEMENT

The authors declare no conflict of interest.

## PREVIOUS PRESENTATION DISCLOSURE

Findings from the present study have not been reported in a previous meeting or published in an abstract.

## REPORTING CHECKLIST DISCLOSURE

This cross‐sectional study has been reported in line with the STROBE Reporting Guidelines.

## Supporting information



Supporting information

## Data Availability

The data are available from the corresponding author upon reasonable request.

## References

[1] McGrath AM , Chen CL , Abrams B , et al. Clinical presentation and outcome in cats with aural squamous cell carcinoma: a review of 25 cases (2010‐2021). J Feline Med Surg. 2022;24:e420‐e432. 36066435 10.1177/1098612X221119144 PMC10812305

[2] London CA , Dubilzeig RR , Vail DM , et al. Evaluation of dogs and cats with tumors of the ear canal: 145 cases (1978‐1992). J Am Vet Med Assoc. 1996;208:1413‐1418. 8635990

[3] Bacon NJ , Gilbert RL , Bostock DE , et al. Total ear canal ablation in the cat: indications, morbidity and long‐term survival. J Small Anim Pract. 2003;44:430‐434. 14582656 10.1111/j.1748-5827.2003.tb00101.x

[4] Pieper JB , Noxon JO , Berger DJ . Retrospective evaluation of ceruminous gland tumors confined to the external ear canal of dogs and cats treated with biopsy and CO(2) laser ablation. J Vet Intern Med. 2023;37:2385‐2390. 37731239 10.1111/jvim.16873 PMC10658490

[5] Thrall DE . Textbook of veterinary diagnostic radiology. Elsevier; 2018. 7th ed.

[6] Fan TM , de Lorimier LP . Inflammatory polyps and aural neoplasia. Vet Clin North Am Small Anim Pract. 2004;34:489‐509. 15062621 10.1016/j.cvsm.2003.10.008

[7] Santagostino SF , Mortellaro CM , Buchholz J , et al. Primary angiocentric/angioinvasive T‐cell lymphoma of the tympanic bulla in a feline leukaemia virus‐positive cat. JFMS Open Rep. 2015;1:2055116915593966. 28491370 10.1177/2055116915593966 PMC5362019

[8] de Lorimier LP , Alexander SD , Fan TM . T‐cell lymphoma of the tympanic bulla in a feline leukemia virus‐negative cat. Can Vet J. 2003;44:987‐989. 14703086 PMC340369

[9] Abdelgalil AI , Mohammed FF . Clinical, ultrasonographic and histopathological diagnosis of ceruminous gland tumors in cats. Vet Res Forum. 2021;12:277‐281. 34815837 10.30466/vrf.2020.108341.2569 PMC8576157

[10] Berzon JLBS . Recurrent otitis externa‐media secondary to a fibroma in the middle ear. J Am Anim Hosp Assoc. 1980;980:73‐77.

[11] Greci V , Mortellaro CM . Management of otic and nasopharyngeal, and nasal polyps in cats and dogs. Vet Clin North Am Small Anim Pract. 2016;46:643‐661. 26947114 10.1016/j.cvsm.2016.01.004

[12] Rogers KS . Tumors of the ear canal. Vet Clin North Am Small Anim Pract. 1988;18:859‐868. 3264960 10.1016/s0195-5616(88)50086-4

[13] Cuschieri S . The STROBE guidelines. Saudi J Anaesth. 2019;13:S31‐S34. 30930717 10.4103/sja.SJA_543_18 PMC6398292

[14] De Magistris AV , Rossi F , Valenti P , et al. CT features of gastrointestinal spindle cell, epithelial, and round cell tumors in 41 dogs. Vet Radiol Ultrasound. 2023;64:271‐282. 36382620 10.1111/vru.13188

[15] Lee S , Jang Y , Lee G , et al. CT features of malignant and benign oral tumors in 28 dogs. Vet Radiol Ultrasound. 2021;62:549‐556. 34236121 10.1111/vru.12996

[16] Tanaka T , Akiyoshi H , Mie K , et al. Contrast‐enhanced computed tomography may be helpful for characterizing and staging canine gastric tumors. Vet Radiol Ultrasound. 2019;60:7‐18. 30123960 10.1111/vru.12677

[17] Fukuda S , Kobayashi T , Robertson ID , et al. Computed tomographic features of canine nonparenchymal hemangiosarcoma. Vet Radiol Ultrasound. 2014;55:374‐379. 24382330 10.1111/vru.12136

[18] Fukushima K , Kanemoto H , Ohno K , et al. CT characteristics of primary hepatic mass lesions in dogs. Vet Radiol Ultrasound. 2012;53:252‐257. 22244075 10.1111/j.1740-8261.2011.01917.x

[19] Nishino M , Hayakawa K , Minami M , et al. Primary retroperitoneal neoplasms: cT and MR imaging findings with anatomic and pathologic diagnostic clues. Radiographics. 2003;23:45‐57. 12533639 10.1148/rg.231025037

[20] Leela‐Arporn R , Ohta H , Shimbo G , et al. Computed tomographic features for differentiating benign from malignant liver lesions in dogs. J Vet Med Sci. 2019;81:1697‐1704. 31597816 10.1292/jvms.19-0278 PMC6943317

[21] Lamb CR , Sibbing K , Priestnall SL . Pathologic basis for rim enhancement observed in computed tomographic images of feline nasopharyngeal polyps. Vet Radiol Ultrasound. 2016;57:130‐136. 26763944 10.1111/vru.12335

[22] Belmudes A , Pressanti C , Barthez PY , et al. Computed tomographic findings in 205 dogs with clinical signs compatible with middle ear disease: a retrospective study. Vet Dermatol. 2018;29:45. 28994490 10.1111/vde.12503

[23] Oliveira CR , O'Brien RT , Matheson JS , et aluted tomographic features of feline nasopharyngeal polyps. Vet Radiol Ultrasound. 2012;53:406‐411. 22548247 10.1111/j.1740-8261.2012.01931.x

