# Supplementary material for: Computed tomographic findings in 25 cats with ear canal neoplasia
Source: Vet Radiol Ultrasound. 2024 Dec 16;66(1):e13467. doi: 10.1111/vru.13467 (PMC11649876; doi:10.1111/vru.13467)
Supplement: Supplementary file 1 — Supporting information [file VRU-66-0-s001.docx]

**Table S1** Image analysis form

| A1: Is there focal or multifocal tissue enlargement (i.e., a mass or masses) present?  1.     Yes  2.     No  A2: If a mass is present, is it single or multiple?  1. Single  2. Multiple (unilateral)  3. Multiple (bilateral)  4. NA (no mass identified) |
| --- |
| A3: On which side is the tissue enlargement most severe? For cases in which focal or multifocal tissue enlargement is not identified, please select the answer choice with the most significant abnormalities.  1. Right  2. Left  For the following items, please reference only the largest mass.  Please regard the side with the largest mass as “ipsilateral”.    B1: Which description best fits the shape of the mass?  1.     Round or ovoid, broad-based  2.     Round or ovoid, pedunculated  3.     Round or ovoid, cannot tell if broad-based or pedunculated  4.     Plaque-like (diffusely infiltrative along ear canal)  5.     Amorphous  6.     Does not fit in above categories (please explain in comments)  7.     NA (no mass identified) |
| B2: With which structure is the center of the mass most closely associated?  1.     Vertical ear canal  2.     Horizontal ear canal  3.     Middle ear  4.     Inner ear  5.     Unable to categorize (describe in comments)  6.     NA (no mass identified) |
| B3: Does the mass involve the vertical ear canal?  1. Yes - compression/displacement  2. Yes – invasion to mild destruction  3. Yes – moderate to severe destruction  4. Yes – both compression/displacement and invasion/destruction  5. No  6. NA (no mass identified) |
| B4: Does the mass involve the horizontal ear canal?  1. Yes - compression/displacement  2. Yes – invasion to mild destruction  3. Yes – moderate to severe destruction  4. Yes – both compression/displacement and invasion/destruction  5. No  6. NA (no mass identified)  B5: Does the mass involve the middle ear?  1. Yes - compression/displacement  2. Yes – invasion to mild destruction  3. Yes – moderate to severe destruction  4. Yes – both compression/displacement and invasion/destruction  5. No  6. NA (no mass identified)  B6: Does the mass involve the inner ear?  1. Yes - compression/displacement  2. Yes – invasion to mild destruction  3. Yes – moderate to severe destruction  4. Yes – both compression/displacement and invasion/destruction  5. No  6. NA (no mass identified)  C1: What is the lesion attenuation uniformity of the mass?  1.     Homogeneous  2.   Heterogeneous  3.   NA (no mass identified)  C2: Is there intralesional gas?  1. Yes  2. No  3. NA (no mass identified)  C3: Is there intralesional fat?  1. Yes  2. No  3. NA (no mass identified)  C4: Is there intralesional mineral? 1. Yes 2. No 3. NA (no mass identified)  C5: Is there intralesional fluid? 1. Yes 2. No 3. NA (no mass identified)  C6: Is there contrast enhancement of the mass? 1.     Yes – diffusely heterogeneous 2.     Yes - heterogeneous with peripheral enhancement (centrally sparing) 3.     Yes - homogeneous  4.     No  5.     NA (no mass identified)  D1: Is there ipsilateral bony lysis? (Lysis is defined as destruction of cortical or trabecular architecture) 1.     Yes - tympanic bulla 2.     Yes - calvarium 3.     Yes - other (describe in comments) 4.     Yes - combination of the above (list in comments) 5.     No  D2: Is there ipsilateral periosteal proliferation? 1.     Yes - aggressive (including amorphous, sunburst, spiculated, palisading, columnar) 2.     Yes - nonaggressive (including smooth lamellar) 3.     No  D3: Is there ipsilateral enlargement of skull foramina? 1.  Yes 2.  No  D4: Is there enhancement of the adjacent meninges? 1.   Yes 2.   No  D5: Is there involvement of other intracalvarial tissues? 1.     Yes - compression  2.     Yes - invasion  3.     Yes - compression and invasion 4.     No  D6: Is the ipsilateral parotid salivary gland affected? 1. Yes - compression/displacement 2. Yes - invasion/destruction 3. Yes – combination of the above 4. No 5. NA (No mass identified)  D7: Is the ipsilateral surrounding musculature affected? 1. Yes - compression/displacement 2. Yes - invasion/destruction 3. Yes - atrophy 4. Yes - combination of the above (list in comments) 5. No 6. NA (No mass identified)  D8: Is the adjacent pharynx affected? 1. Yes - compression/displacement  2. Yes - invasion/destruction 3. Yes – combination of the above 4. No 5. NA (No mass identified)  For the following questions, in cases where a mass is not identified, regard “ipsilateral” as the side you selected in question A3.   E1: Is there fluid accumulation within the ipsilateral ear canal? (Fluid defined as non-contrast enhancing soft tissue to fluid attenuating material) 1.     Yes - mild (<50% of horizontal ear canal) 2.     Yes - moderate (>50% of horizontal ear canal) 3.     Yes - both horizontal and vertical ear canal 4.     No  E2: Is there debris within the ipsilateral ear canal?  1.     Yes - mild (<50% of horizontal ear canal) 2.     Yes - moderate (>50% of horizontal ear canal) 3.     Yes - both horizontal and vertical ear canal 4.     No  E3: Is mineralization of the ipsilateral auricular and/or annular cartilages present? 1.     Yes - mild/moderate 2.     Yes - severe 3.     No  E4: Is there ipsilateral external ear canal thickening and/or folding? (The rationale for this question is to capture evidence of otitis externa in addition to the mass that has been evaluated above) 1.     Yes - thickening only 2.     Yes - thickening and folding  3.     No  E5: Is there ipsilateral luminal stenosis of the external ear canal? (The rationale for this question is to capture evidence of otitis externa in addition to the mass that has been evaluated above) 1.     Yes  2.     No  E6: Is there mucosal thickening of the walls of the ipsilateral tympanic cavity? 1.     Yes 2.     No  E7: Is there ipsilateral tympanic cavity effusion? 1.     Yes 2.     No  E8: Is there ipsilateral tympanic bulla thickening? 1.     Yes 2.     No  E9: Is there ipsilateral expansion of the tympanic cavity? 1. Yes 2. No  F1: Is there fluid accumulation within the contralateral canal? (Fluid defined as non-contrast enhancing soft tissue to fluid attenuating material) 1.    Yes - mild (<50% horizontal ear canal) 2.     Yes - moderate (>50% of horizontal ear canal) 3.     Yes - both horizontal and vertical ear canal 4.     No  F2: Is there debris within the contralateral canal?  1. Yes - mild (<50% horizontal ear canal) 2. Yes - moderate (>50% of horizontal ear canal) 3. Yes - both horizontal and vertical ear canal 4. No  F3: Is mineralization of the contralateral auricular and/or annular cartilages present? 1.    Yes - mild/moderate 2.     Yes - severe 3.     No  F4: Is there contralateral external ear canal thickening and/or folding?  (The rationale for this question is to capture evidence of otitis externa in addition to the mass that has been evaluated above) 1.    Yes - thickening only 2.     Yes - thickening and folding  3.     No  F5: Is there contralateral luminal stenosis of the external ear canal? 1.    Yes  2.     No  F6: Is there mucosal thickening of the walls of the contralateral tympanic cavity? 1.    Yes 2.     No  F7: Is there contralateral tympanic cavity effusion? 1.     Yes 2.     No  F8: Is there contralateral tympanic bulla thickening? 1.     Yes 2.     No  F9: Is there contralateral expansion of the tympanic cavity? 1. Yes 2. No  G1: Are the parotid lymph nodes enlarged? (subjective assessment) 1.     Yes - unilateral, ipsilateral 2.     Yes - unilateral, contralateral  3.     Yes - bilateral  4.     No 5.     Not identified (but expected location included in the field-of-view) 6.     Expected location not included in the field of view  G2: Are the lateral retropharyngeal lymph nodes enlarged? (subjective assessment) 1.     Yes - unilateral, ipsilateral 2.     Yes - unilateral, contralateral  3.     Yes - bilateral  4.     No 5.     Not identified (but expected location included in the field-of-view) 6.     Expected location not included in the field of view  G3: Are the medial retropharyngeal lymph nodes enlarged? (subjective assessment) 1.     Yes - unilateral, ipsilateral 2.     Yes - unilateral, contralateral  3.     Yes - bilateral  4.     No 5.     Not identified (but expected location included in the field-of-view) 6.     Expected location not included in the field of view  G4: Are the mandibular lymph nodes enlarged? (subjective assessment) 1.     Yes - unilateral, ipsilateral 2.     Yes - unilateral, contralateral  3.     Yes - bilateral  4.     No 5.     Not identified (but expected location included in the field-of-view) 6.     Expected location not included in the field of view  G5: Are the superficial cervical lymph nodes enlarged? (subjective assessment) 1.     Yes - unilateral, ipsilateral 2.     Yes - unilateral, contralateral  3.     Yes - bilateral  4.     No 5.     Not identified (but expected location included in the field-of-view) 6.     Expected location not included in the field of view  G6: Are the deep cervical lymph nodes enlarged? (subjective assessment) 1.     Yes - unilateral, ipsilateral 2.     Yes - unilateral, contralateral  3.     Yes - bilateral  4.     No 5.     Not identified (but expected location included in the field-of-view) 6.     Expected location not included in the field of view |
